# Effect of Biochar on the Production of L-Histidine From Glucose Through *Escherichia coli* Metabolism

**DOI:** 10.3389/fbioe.2020.605096

**Published:** 2021-01-07

**Authors:** Yang E, Jun Meng, Heqing Cai, Caibin Li, Sainan Liu, Luming Sun, Yanxiang Liu

**Affiliations:** ^1^Liaoning Biochar Engineering & Technology Research Center, Shenyang Agricultural University, Shenyang, China; ^2^Guizhou Tobacco Company in Bijie Company, Bijie, China

**Keywords:** biochar, L-histidine, *E. coli*, biosynthesis, organic compounds

## Abstract

The organic compounds from biochar play a role of hormone analogs, stimulating the expression of metabolites by controlling related gene and protein. In this experiment, we reported the L-histidine biosysthesis was promoted by biochar treatment in *E. coli* unlike genetic engineering of the traditional method. The related results indicated the most optimal concentration was found to be 3%, and 7% is the lethal dose. *E. coli* was inhibited in the high-concentration treatment. On the other hand, docking technology was usually used as drug screening, basing on Lock-and-key model of protein in order to better understand mechanisms. So the organic compounds of biochar from GC-MS analysis that acted as ligands were connected to HisG protein controlling L-histidine biosysthesis in *E. coli*. The result showed that the three organic molecules interacted with HisG protein by hydrogen bond. So we considered that these three compounds play regulatory roles in L-histidine biosysthesis, and the *his*G *gene* expression fully supports this conclusion.

## Introduction

L-histidine is an essential proteinogenic amino acid in plants and animals. Therefore, amino acids are widely used in the medical and agriculture industries. Many studies have indicated that several diseases are related to a lack of histidine (Kulis-Horn et al., 2014). Appropriate histidine levels in the diet (12 mg) can effectively prevent obesity and metabolic disorders (Kasaoka et al., 2004; Tuttle et al., 2012). In cancer therapy, all methods focus on enhancing the body’s natural defense mechanisms (Maus et al., 2014). Recent studies have shown that suitable dietary supplementation of histidine can boost the effectiveness of the immune system (Kanarek et al., 2018).

The traditional biosynthetic pathway of histidine includes 10 enzymatic reactions that convert phosphoribosyl-1-pyrophosphate into histidine (Lazcano et al., 1996; Ikeda, 2003). The process mainly depends on the degradation of natural protein resources. There are many challenges in increasing histidine content. Therefore, artificially synthesized histidine is being developed rapidly (Shibasaki et al., 2008; Mitsuhashi, 2014). However, the chemical synthesis of histidine produces a racemic mixture with unnatural compounds, which may not be beneficial for health. As a result, administrative agencies have ruled that histidine production is not up to standard. Therefore, microbial producers and fermentation processes have become the focus of research.

Agricultural processes produce waste in massive amounts (Shi, 2011). This waste contains large amounts of fiber and lignin. Agricultural biomass is subjected to thermal treatment under an oxygen-limited atmosphere, yielding a carbon-rich, solid product known as biochar (Lehmann, 2009; Meng, 2013). Many different biomass and preparation processes can be used to produce biochar for different purposes. However, there is a challenge in obtaining suitable biochar precursors for specific purposes. Current research suggests that biochar can provide a suitable environment for plants and microorganisms (Yang et al., 2015). The most notable functions of biochar are (1) sorption in both organic and inorganic compounds, (2) changing the cation exchange capability, (3) providing hormone analogs for plants, (4) altering soil pH, and (5) providing a place and irritant substance for microbial growth (Chan et al., 2007; Yang et al., 2019; Hong et al., 2020). Biochar contains several organic compounds with various biological functions. For example, 2-Acetyl-5-methylfuran from biochar can promote rice seedling growth by playing the role of a hormone analog (Yang et al., 2019). Moreover, Yuan et al. (2017) reported that organic molecules from biochar, including 14 candidate compounds, have a positive effect on the cold tolerance of rice seedlings. Many studies have reported that biochar can benefit microbial growth (Lehmann, 2009). The traditional biosynthetic pathway of L-histidine mainly depends on bacteria, such as *Salmonella typhimurium*, *Escherichia (E.) coli*, and *Corynebacterium glutamicum* (Lazcano et al., 1996; Jung et al., 2010). A previous study indicated that biochar could change the microbial community (Yang et al., 2015). However, there is no reports that the biochar can promote the bacteria producing the L-histidine. Therefore, *E. coli* was selected as a representative bacterium of the biosynthetic pathway in L-histidine in order to study influence of biochar on bio-synthesis of L-histidine. On the other hands, we consider that there are compounds from biochar promoting the bio-synthesis of L-histidine. However, it is difficult to find out the compounds through experiments due to the composition complexity of biochar. So, we hypothesize that the organic compounds in biochar could interact with receptor protein controlling L-histidine. If the organic compounds that acted as ligands was connected to the active site of receptor protein and the same mechanism of action as ligands, the organic compounds played the analogous biological function. So the autodocking is employed to select the compounds of biochar which can promote the bio-synthesis of L-histidine from theory basing on lock-and-key model of protein in order to efficiently produce L-histidine from *E. coli* in biochar and to understand the compounds from biochar promoting biosynthetic L-histidine of mechanism. The result provides another pathway for the production of L-histidine.

## Materials and Methods

### Biochar Preparation and Culture Conditions

Agricultural biomass from tobacco straw was used in this study. All biomass was obtained from the Bijie tobacco company in China. The raw materials were pyrolyzed at 400°C for 30 min at a rate of ∼15°C/min. Biochar was generated at 300, 400, 500, 600, and 700C∘ in the preliminary experiment. However, the biochar at 400°C was derived for the treatments in this study, as this leads to the most favorable L-histidine content.

*E. coli* was obtained from Liaoning Biochar Engineering & Technology Research Center and cultivated in Luria-Bertani medium according to a previously reported method (Chu et al., 2020). The inducers include 2 g/L L-arabinose, 15 g/L agar powder, 5 g/L NaCl and 5 mol/L NaOH for pH 7.0. The biochar was then added to the medium at concentrations of 0% (control), 1, 3, 5, and 7% (inducers: biochar = w: w).

### Biochar Characterization

The physicochemical properties of the biochar were analyzed in order to define the materials present (Table 1). Aqueous extracts of biochar (1:10 w:w) were prepared in MilliQ ultrapure water (Millipore, United States) for 1 h. The extract liquors were checked for pH, electrical conductivity, N-NO_3_, and N-NH_4_ (AA3, SEAL, Germany). The nutrient contents of biochar (including K, Na, Mg, Ca, Cu, Fe, Zn, B, and P) were tested using an atomic absorption spectrometer (AA6880, Shimadzu, Japan). An element analyzer (VARIO MACRO CUBE, Elementder, Germany) was employed to analyze the total organic carbon in the biochar.

**TABLE 1:** Physicochemical properties of biochar-exacted liquor.

**Element**	**Biochar concentration**			
	**1%**	**3%**	**5%**	**7%**
K	3.112	3.131	3.217	3.227
Na	3.063	3.072	3.188	3.195
Mg	0.029	0.031	0.033	0.036
Ca	0.026	0.025	0.038	0.041
Zn	0.000	0.000	0.000	0.000
P	0.002	0.002	0.003	0.003
S	0.607	0.611	0.625	0.628
N-NO_3_	0.0002	0.0004	0.0004	0.0005
N-NH_4_	0.0000	0.0000	0.0000	0.0000
pH	7.55	7.60	7.71	7.82
EC (μs/cm)	10.3	11.1	12.2	13.2
Total organic carbon	49.3	51.5	52.1	56.8

*The element unit is g/kg.*

### Analysis of Organic Compounds From Biochar by GC-MS

The carbon skeleton is not absorbed by microorganisms. Small organic compounds can enter the cytomembrane, sometimes affecting microbial growth. Therefore, different polar organic solvents were employed to extract the organic compounds from biochar. Biochar (1.5 g) was homogenized with 100 mL non-polar organic solvents (heptane and hexane) or polar organic solvents (methanol, ethanol, acetonitrile, chloroform, ethyl acetate, and dichloromethane). The analysis procedure was performed according to a previous study (Yang et al., 2019). The ionization efficiency and the MS response were periodically tested by 1 μL mL^–1^ IPA (1,000 μL mL^–1^ in methylene) as certified reference material (Profumo et al., 2020) in order to assure the quality of related data.

### Molecular Docking Analysis

The molecular structures of the organic compounds from the GC-MS results were constructed by Gaussview. Then, Gaussian 09 was used for geometric optimization in order to determine the stable structure (B3LYP functional with 6-311 + G). The HisG (ID: 1H3D) protein structure was downloaded from the Research Collaboratory for Structural Bioinformatics (RCSB) protein database. AutoDockTools (ADT) were used to prepare the ligands and 1H3D receptor and to determine the “search space.” Organic compound optimization was docked with 1H3D in order to identify molecules that can promote the biosynthesis of L-histidine.

### Gene Expression Analyses

ATP phosphoribosyltransferase (HisG), which catalyzes the first step of histidine biosynthesis, is the most important enzyme regulated at the enzymatic level. Therefore, in this study, *his*G gene expression was analyzed to understand the synthesis state of L-histidine. DNA from the culture medium was isolated from 0.5 g using nucleic acid reagent (Takara accompany, Dalian, China) following the manufacturer’s instructions. DNA quality and concentration were tested by gel electrophoresis and a NanoDrop spectrophotometer (NanoDrop Technologies, Wilmington, DE, United States). The *his*G primers were obtained according to a previous paper (18 S FWD: GTGCCAGCAGCCGCGGTA; 18 S RV: TGGACCGGCCAGCCAAGC; Huada Gene Technology Company, Shenzhen, China). Quantitative RT-PCR (qRT-PCR) was carried out in a 10-μL reaction vessel containing 5 μL 2.5 × RealMaster Mix, 20 × SYBR solution (Takara accompany, Dalian, China), 0.2 μL of both forward and reverse primers, and 1 μL of diluted cDNA (1:10). PCR amplification was performed using System LightCycler 480 equipment (Roche Applied Science, Germany); the qRT-PCR procedure comprised 95°C for 1 min, followed by 40 cycles of 95°C for 10 s, 55°C for 30 s, and 68°C for 1 min. Values for gene expression were calculated following the method outlined by Rieu and Powers (2009) using delta-delta Ct.

### Substrate and Product Analyses

The glucose concentration was measured using an SBA-40E biological sensor (Shandong Academy of Sciences, Shandong, China). L-histidine was analyzed by UPLC (I-Class; Waters Company, United States) coupled to a UV photodiode array detector. And a Discovery C-18 column (2.1 × 50 mm, 1.7 μm; Waters Company, United States) was used in this experiment. The protocol for L-histidine was adapted from Ning et al. (2016). The sample manager FTN was maintained at 10°C. The column of temperature was heated 40°C. The 0.1% formic acid in milliQ water was used as the mobile phase at 0.6 mL/min. And then, 0.2 μL samples were run for 5 min, the UV photodiode array detector was tested at 570 nm, with a data acquisition rate of 20 point/s. An external calibration curve was built in a range between 1 and 10 ng/ml from certified reference of L-histidine (Aladdin accompany, China) though Empower 3 software.

### Statistical Analysis

All numerical data were analyzed using the statistical software Spss (version 26, United States). The addition of ^∗^ and ^∗∗^ indicates that different concentrations of biochar exhibited significant differences at the *P* < 0.05 and *P* < 0.01 levels compared with those of the control, respectively. Three technical and three biological replicates were analyzed for gene expression and *E. coli* inducers.

## Results

### Biochar Has a Positive Impact on the Production of Histidine in *E. coli*

**FIGURE 1:**
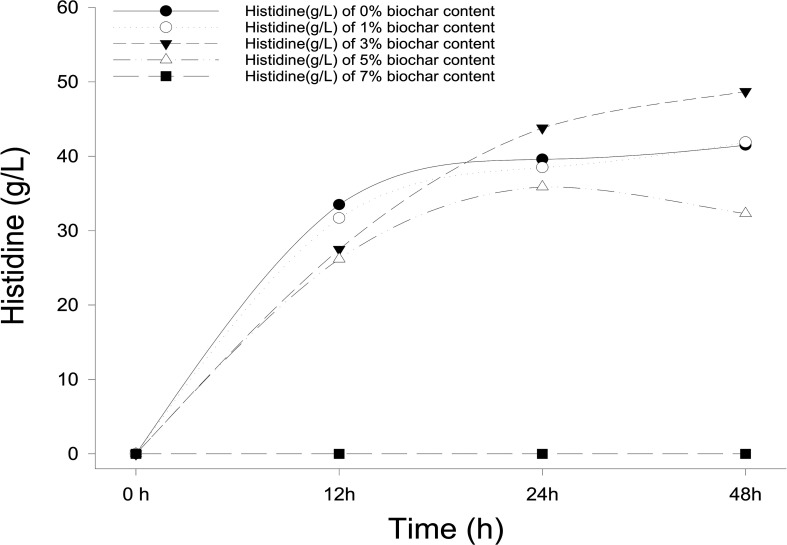
The concentration of L-histidine production in *E. coli* with different biochar concentrations and treatment times.

**FIGURE 2:**
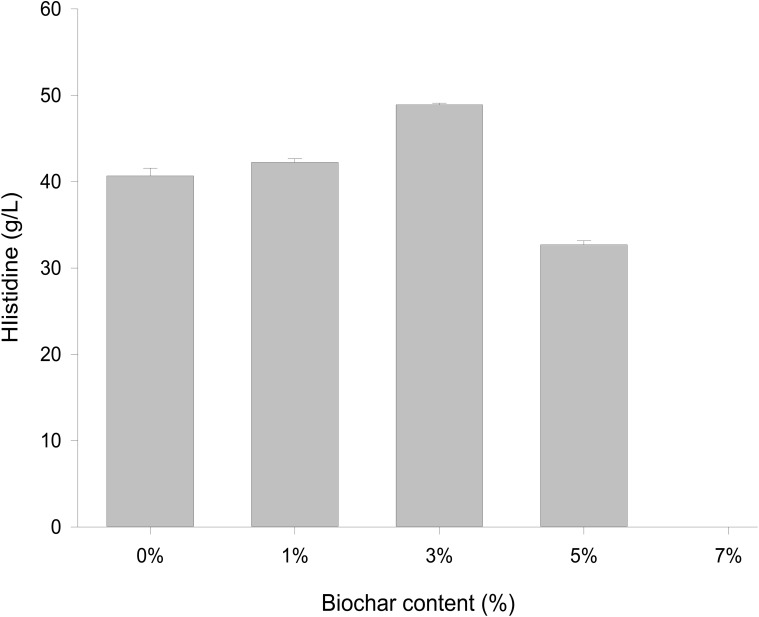
The concentration of L-histidine production with different biochar treatments after 48 h.

Different concentrations of biochar (0, 1, 3, 5, and 7%) were studied in the same *E. coli* culture environment (Figure 1). L-histidine was promoted in all concentrations, except for the 7% biochar treatment (0 g/L L-histidine concentration). Histidine production under 3% biochar treatment, which reached about 48.7 g/L (Figure 2, *P* < 0.05), was significantly greater than that of the control, 1%, and 5% treatments after 48 h of culture. Variations in L-histidine production among the different biochar treatments resulted in different degrees of inhibition. With 12 h of treatment, the degree of inhibition was lower in all biochar-treatment groups compared with that of the control. Higher biochar concentrations inhibited L-histidine production in *E. coli* more strongly. However, inhibition was eliminated after 24 h with 3% biochar treatment. These results indicated that at lower concentrations, *E. coli* was adapted for the biochar environment (Figure 1). Over time, *E. coli* produced greater amounts of L-histidine in low concentrations of biochar (1 and 3%). However, the high biochar concentrations (5 and 7%) did not eliminate inhibition. In the 7% biochar treatment, L-histidine was completely inhibited. The results showed that 7% biochar was lethal in *E. coli.* On the other hand, many different elements and organic compounds from biochar provided nutrients for the microorganisms (Table 1). In addition, the microorganisms were able to live on the special structure of biochar. These factors benefited *E. coli* survival. The L-histidine concentration varied with different biochar concentrations.

### Analysis of Organic Compounds in Biochar

**FIGURE 3:**
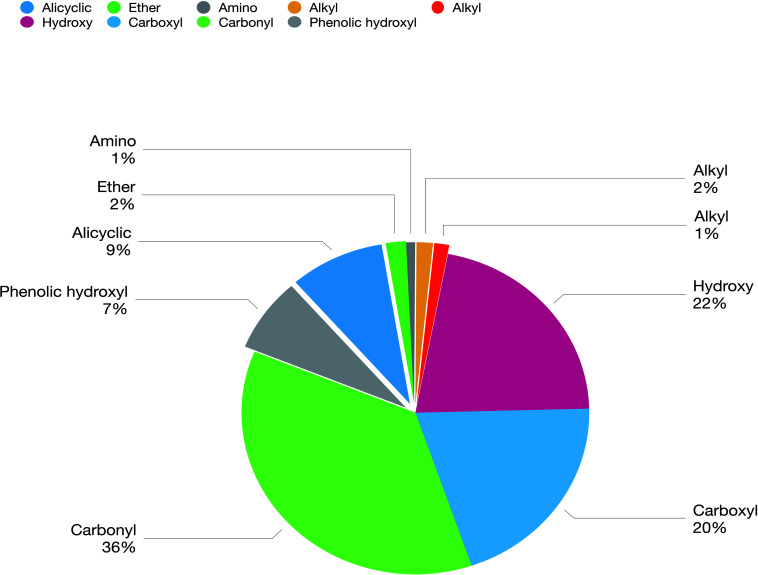
Composition and concentration of functional groups in the organic molecules from biochar-extraction solution.

The biochar comprised a carbon skeleton, inorganic elements (including N, P, and K), ash content, and organic compounds. In plants and microorganisms, the inorganic elements and organic compounds from biochar are absorbed, which affect their growth and metabolites. L-histidine is the metabolic product of *E. coli*. Therefore, in the following experiment, the influence of organic compounds was studied. There are many unknown organic compounds in biochar that are difficult to analyze. However, compound data in GC/MS was used to facilitate the identification of these unknowns. In this experiment, ion trap MS was employed to analyze the products of biochar extracts using polar and non-polar solvents. Nine different functional groups, which contribute to different biological functions, were found in the organic compounds (Figure 3). Cartonyl, carboxyl, and hydroxy were the main functional groups in the biochar-extraction solution (78%).

### Organic Compounds Promoting Histidine Biosynthetic Gene Expression

**FIGURE 4:**
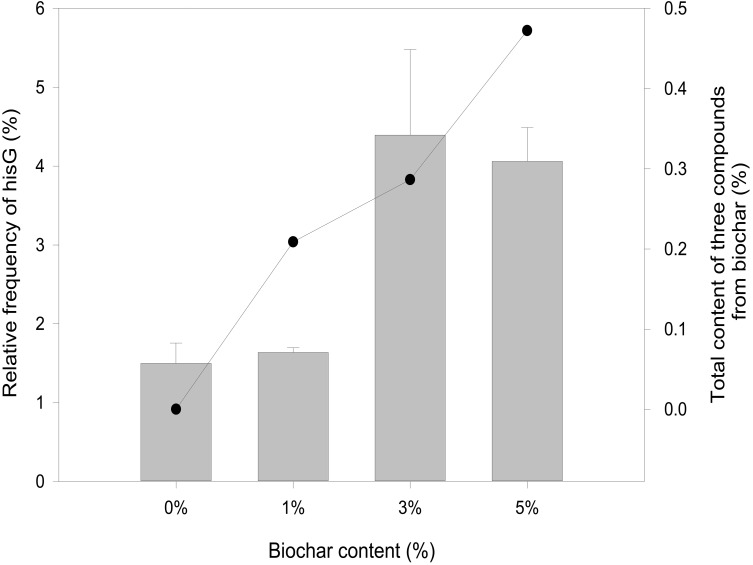
Quantitative RT-PCR analysis of *his*G gene expression from 0, 1, 3, and 5% biochar treatment for 48 h (bar graph). The sum of eugenol, salicyl alcohol, and cyclopentane from biochar over a 48-h period.

Overexpression of a rate-limiting enzyme is one of the most common strategies to improve end-product accumulation. The HisG protein plays an important role in histidine metabolic process. The *his*G gene was analyzed to define the molecular changes resulting from different biochar treatments; its expression was suppressed in the control and each concentration of biochar treatment (Figure 4). There was no significant difference in the control or the 1% biochar concentration. *E. coli* treated with 3% biochar exhibited the highest expression of *his*G, and the gene was inhibited at a 7% biochar concentration. The observations of *his*G gene expression were consistent with the L-histidine product results (Figure 2). Thus, the analysis indicated that *his*G expression along with the L-histidine product can respond to compounds in the biochar.

### Docking Analysis

**FIGURE 5:**
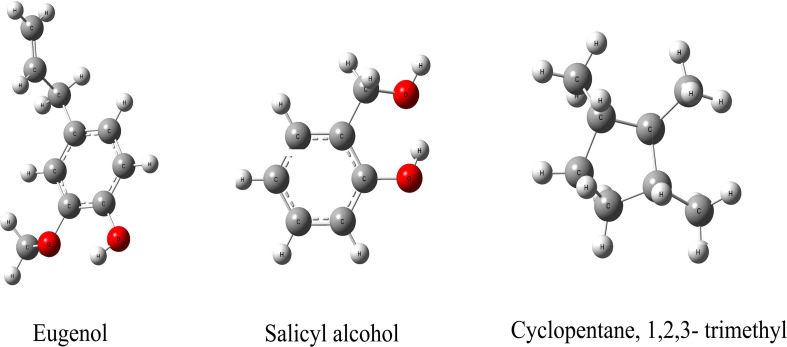
The molecules from biochar that could interact with the HisG protein.

**FIGURE 6:**
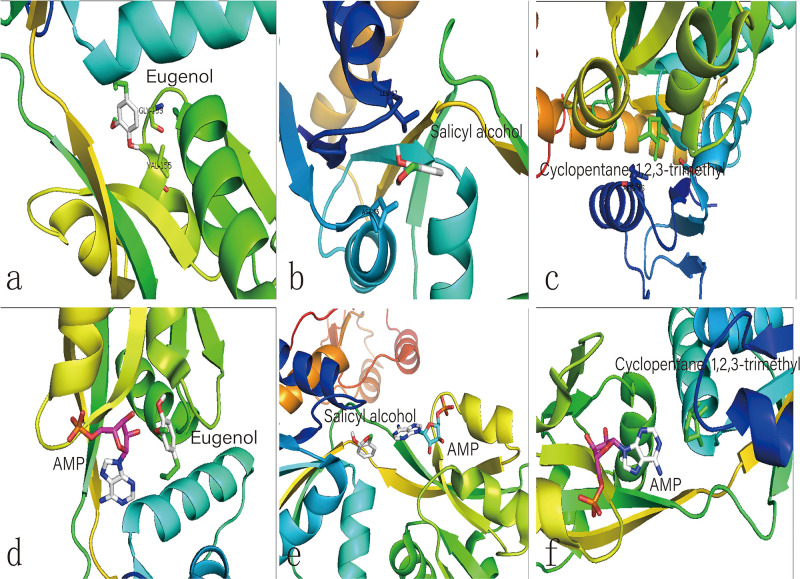
Molecular docking analysis of HisG protein. **(a–c)** illustrate the interaction of eugenol, salicyl alcohol, and 1,2,3-trimethylcyclopentane with the activity site of the HisG protein; Comparison between AMP and eugenol **(d)**, salicyl alcohol **(e)**, and cyclopentane **(f)** in the HisG protein activity site.

We hypothesize that the biochar including organic compounds can interact with protein receptor (i.e., L-histidine biosysthesis of regulatory protein, hisG protein). The molecules of biological function that act as ligands are connected to hisG protein. Therefore, the hisG protein structure involved in L-histidine biosysthesis from *E. coli* was download from RCSB, the result indicated that three candidate modules (Figure 5) acted as ligands for target proteins (Figure 6). The biochar included different types of organic compounds (Figure 3), but there are several organic compounds including biological function. On the other hand, the three candidate molecules content is a little. So we considered that the organic compounds from biochar played a role of hormone analogs that controlled the metabolic process of microorganism. The related gene was expressed in the organic compounds stimulating (Figure 4).

## Discussion

In our study, the *his*G *gene* expression was induced by different biochar treatment, including the promotion of L-histidine biosynthesis from *E. coli*. Total L-histidine contents in 0% as well as 1, 3, and 5% treatments compared to 7% treatment in this experiment. The results indicated that the low biochar treatment was benefit for L-histidine biosynthesis. However, L-histidine biosynthesis was inhibited in the high biochar treatment. So, we considered that some organic compounds could control the L-histidine biosysthesis in the *E. coli* cell.

The metabolic process of L-histidine in *E. coli* was complex and controlled by genes, proteins, or other compounds (Martin et al., 2017). Therefore, it was difficult to understand completely (Zhao et al., 2015). Genomics, proteomics, and metabolomics are usually employed to discuss the influence of allogenic material (Lal et al., 2018). However, it was difficult to understand the interaction between allogenic material and molecules from microorganisms. When the allogenic mixture was mixed, the analysis became more difficult. And then, it was also difficult to understand whether the precise compounds from biochar could affect *his*G expression and biosysthesis by experiment method (Kulis-Horn et al., 2014). Therefore, AutoDock dockings were employed to explore molecules from biochar organic compounds (Ciemny et al., 2018). The result indicated three candidate molecules (Figure 5) could interact with a favorable protein site, but the combined site slightly differs. Because these three organic compounds could potentially affect the HisG protein, the relationship between *his*G *gene* expression and the concentrations of the three compounds was analyzed. The area-normalization method was employed to calculate the compound concentrations (Roy et al., 2018). The amount of different compounds in the biochar was highest under 3% biochar treatment. Therefore, the sum of the three organic compounds was reflected in the relationship between the compounds and the *his*G *gene* (*r* = 0.459, *p* = 0.003). The results indicated that the *his*G *gene* increased with the sum of the three organic compounds from biochar (Figure 4), indicating that these three compounds could stimulate *his*G *gene* expression.

The same site of action was observed in the HisG protein (Figures 6e–g). The hydrogen bond was mainly a chemical combination between the three molecules and the activity site of HisG proteins such as AMP. In eugenol, the molecule could form a hydrogen bond with GLY153 and VAL-155 from protein residues (Figure 6a), salicyl alcohol was combined with LEU-17 and ASP-55 protein residues (Figure 6b), and 1,2,3-trimethylcyclopentane, interacted with ARG16 and ASP-55 protein residues (Figure 6c). The results showed that there were different acting sites with protein residues, but the acting area was the same. Therefore, it was speculated that the HisG protein was controlled by the three molecules. The results indicated that biochar could induce L-histidine biosysthesis in *E. coli*. The process of molecules interacting with HisG protein is illustrated in Cartoon1–3.

The concentrations of the three compounds, along with other organic compounds, were fairly low in the biochar. However, the molecules could induce the L-histidine biosysthesis of *E. coli* at a rate 17.3% higher than that of the control group (*r* = 0.756, *p* = 0.005). Therefore, this experiment shows that the molecules may play roles as hormone analogs, which affect plant growth, microbial structure, and the concentrations of metabolic substances (Yang et al., 2015, 2019). Therefore, organic compounds warrant consideration in the research field of biochar.

## Conclusion

Compared with traditional genetic engineering methods to produce L-histidine, we found the L-histidine biosysthesis increased with an increasing concentration of biochar treatment (1, 3, and 5%), excepting 7% or higher concentration. According to molecular docking, three organic compounds could interact with HisG protein, involving in the response to L-histidine biosysthesis. The compounds acted as hormone analogs. It’s easy to explain that the *his*G *gene* expression were positively correlated.

## Data Availability Statement

The raw data supporting the conclusions of this article will be made available by the authors, without undue reservation.

## Author Contributions

YE and JM: data curation, funding acquisition, writing—original draft, and writing—review and editing. JM: funding acquisition and supervision. YL: funding acquisition, methodology, and resources. SL and LS: formal analysis and methodology. CL and HC: validation and visualization. All authors contributed to the article and approved the submitted version.

## Conflict of Interest

The authors declare that the research was conducted in the absence of any commercial or financial relationships that could be construed as a potential conflict of interest.
